# Vitamin D Concentration during Early Pregnancy and Adverse Outcomes among HIV-Negative Women in Dar-es-Salaam, Tanzania: A Case-Control Study

**DOI:** 10.3390/nu11122906

**Published:** 2019-12-02

**Authors:** Aneth V. Kalinjuma, Anne Marie Darling, Christopher R. Sudfeld, Ferdinand Mugusi, Julie Wright, Ajibola I. Abioye, Said Aboud, Chloe McDonald, Ellen Hertzmark, Kevin C. Kain, Wafaie W. Fawzi

**Affiliations:** 1 Intervention and Clinic Trials Department, Ifakara Health Institute, P.O. Box 53, Ifakara, Morogoro, Tanzania; 2 Department of Global Health and Population, Harvard T.H. Chan School of Public Health, Boston, MA 02115, USA; 3 Departments of Internal Medicine; and Microbiology and Immunology, School of Medicine, Muhimbili University of Health and Allied Sciences, P.O. Box 65001, Dar-es-Salaam, Tanzania; 4 Department of Medicine, Tropical Disease Unit, University Health Network—Toronto General Hospital, University of Toronto, Toronto, ON M5G 1L7, Canada

**Keywords:** first trimester, pregnant women, vitamin D concentration, stillbirth, premature, small for gestational age, non-linear

## Abstract

We examined the associations of plasma vitamin D concentration and adverse pregnancy outcomes among HIV-negative women in Dar-es-Salaam, Tanzania. We used an unmatched case-control study design, with 25-hydroxyvitamin D [25(OH)D] concentration assessed in the first trimester. Cases were individuals with adverse pregnancy outcomes, including stillbirth, premature birth, or small for gestational age births (SGA). Unconditional logistic regression and weighted logistic regression models were used to describe the associations of 25(OH)D concentration with the composite of adverse pregnancy outcome and individual adverse pregnancy outcomes, respectively. We included 310 cases and 321 controls. In controls, 5(2%) were vitamin D deficient (25(OH)D < 20 ng/mL), and 17(5%) had insufficient 25(OH)D concentration (20.0–29.9 ng/mL). Women with 25(OH)D < 20 ng/mL had 1.82 times the odds of occurrence of the composite adverse pregnancy outcome (OR = 1.82, 95% CI: 0.56–5.93; *p* = 0.32), however we noted a non-linear association between 25(OH)D concentration and adverse pregnancy outcome (*p* = 0.02). We found a 3-fold increased odds of stillbirth in women with low 25(OH)D concentration (OR = 3.11, 95% CI: 1.18–8.23, *p* = 0.02). Vitamin D concentration in early pregnancy may be an important factor in determining the course of pregnancy. Further research is needed to investigate whether the association of maternal 25(OH)D concentration in early pregnancy and stillbirth is causal.

## 1. Introduction

Adverse pregnancy outcomes are still a major global public health problem. It is estimated that 2.6 million babies were stillborn in 2015 worldwide [1], and that 98% of stillbirths occur in low- and middle-income countries [2]. Prematurity remains a major condition associated with higher infant and child mortality and lifelong disabilities [3,4]. Children who were small for gestational age (SGA) at birth have increased risk of chronic diseases, such as cardiovascular diseases, complications including insulin resistance, and metabolic syndrome [5].

During pregnancy there is an increased demand for micronutrients, including vitamin D, to support fetal growth [6]. Vitamin D has an important role in placental functioning, calcium homeostasis, bone mineralization, muscle contraction, development of the nervous system and cellular function. All these processes are necessary for healthy fetal growth and development [7,8]. Despite its importance, vitamin D deficiency during pregnancy is common, and remains a significant public health problem. High prevalence of vitamin D deficiency in pregnant women has been found in high latitude countries due to limited sunlight and the long winter season [7,8,9,10]. However, in tropical countries where sufficient ultraviolet light is assumed, vitamin D deficiency during pregnancy is also prevalent [11,12,13], possibly related to high skin melanin, aging, skin coverage by clothing, and spending much time in indoors [8,11].

Adverse pregnancy outcomes have been associated with low vitamin D concentrations during pregnancy [7,9,14]. These relationships have been assessed in countries such as the United States of America (USA), China, Bangladesh and the Netherlands [7,10,13,15,16]. In several African settings vitamin D has been examined among Human Immunodeficiency Virus (HIV) infected women [12], with limited assessment among the larger population of HIV-negative women.

Vitamin D concentration may have protective effects during the early period of embryogenesis [7,10,17], with adverse consequences of its deficiency noted later in pregnancy. Few studies have assessed the association of first trimester vitamin D concentration and pregnancy outcomes. This study sought to describe the relationship of first trimester vitamin D concentration with adverse pregnancy outcomes, including stillbirth, premature birth, and SGA birth among HIV-negative women in urban Dar-es-Salaam, Tanzania.

## 2. Materials and Methods

### 2.1. Study Design and Participants

We used an unmatched case-control study design. Cases and controls were selected from pregnant women enrolled between 2010 and 2013 in a randomized double-blind, the trial that assessed oral administration of zinc and vitamin A supplementation starting from early pregnancy (<13 weeks of gestation) on the risk of placental malaria infection and adverse pregnancy outcomes [18]. Briefly, HIV-negative pregnant women in their first or second pregnancy were recruited at their first antenatal care visit or through a demographic surveillance system (DSS) in Temeke district, Dar-es-Salaam. First trimester pregnant women identified though the DSS were referred to the nearest recruitment clinic. In addition to trial supplements, participants received iron and folic acid supplements according to Tanzanian standard of prenatal care. At recruitment, participants provided socio-demographic information, underwent a full clinical examination and provided blood sample for complete blood count test.

Among women from the original trial with samples obtained during the first trimester of pregnancy and having gestational age at delivery 30–42 weeks, known pregnancy outcome, and birth weight (for live birth), we included all cases of stillbirth, prematurity (30–33 completed weeks), and SGA. A random sample of women without these outcomes (controls) was selected from women with live births who delivered at 34–42 weeks of gestation and having infants with birth weight appropriate for gestational age.

### 2.2. Assessment of Vitamin D Status

Plasma 25-hydroxyvitamin D [25(OH)D] concentrations were assessed during early pregnancy (4–13 weeks of gestation). Venous blood (1–2 mL) was collected into an EDTA vacutainer tube and transferred to the laboratory within two hours in a 4 °C container. Specimens were centrifuged for 10 min at 2500 rpm to obtain plasma, which was aliquoted into sterile cryovials and stored at −80 °C freezer in a Clinical Research Laboratory at Muhimbili University of Health and Allied Science (MUHAS), Dar-es-Salaam. The frozen plasma samples were transferred to the University of Toronto, Ontario, Canada for analysis.

Plasma 25(OH)D concentrations were measured by Enzyme Immunoassay (EIA) (Immunodiagnostic Systems Ltd.,Tyne and Wear, United Kingdom) according to the manufacturer’s instructions with 10% of the samples on each plate performed in duplicate. Laboratory technicians were blinded to the study outcomes. The final EIA plates were read by ELISA reader at 405 nm. Concentrations were extrapolated from a 4-parameter non-linear regression curve using Prism7 for Mac OS X software (version 7.0e). The range of detection for 25(OH)D was 6.5 ng/mL to 96.8 ng/mL. One sample (0.2% of all samples) fell below the lower limit of detection and was included at it was read by ELISA reader. There were no samples above the upper limit of detection.

The 25(OH)D concentrations were classified in three ways: (1) Sufficient vitamin D concentration (25(OH)D ≥ 30.0 ng/mL) and low vitamin D concentration (25(OH)D < 30.0 ng/mL), because plasma 25(OH)D concentrations above 30.0 ng/mL are association with beneficial effects on calcium, bone, and muscle metabolism [19]; (2) the Institute of Medicine (IOM) define vitamin D deficiency being the 25(OH)D < 20.0 ng/mL [20]. Therefore we defined sufficient vitamin D concentration as 25(OH)D ≥ 30.0 ng/mL, insufficient vitamin D concentration being 25(OH)D between 20.0–29.9 ng/mL and vitamin D deficient being 25(OH)D ˂ 20.0 ng/mL [19]; and (3) low vitamin D concentration was defined as 25(OH)D < 40.0 ng/mL because the circulating 25(OH)D of at least 40.0 ng/mL is required to support achievement of maximum production of the active hormone 1,25(OH)_2_D in pregnant women [21]. The 25(OH)D concentration < 40.0 ng/mL corresponded to lowest tertile of vitamin D concentration of study participants. We also examined the association of continuous 25(OH)D concentration with the outcomes of interest.

### 2.3. Definition of Cases and Controls

Since vitamin D deficiency may affect stillbirth, early prematurity, and SGA by a common pathway, we used the composite outcome of any of these 3 as our primary outcome. Controls for the primary outcome were women with live birth who delivered at 34–42 weeks of gestation and having infants with birth weight appropriate for gestational age. Stillbirth was defined as fetal demise before birth (after 28 weeks). Early premature birth was defined among live births as occurring at 30–33 weeks of gestational age. The cut-off point of birth <34 weeks was selected to minimize the likelihood of misclassification of prematurity as gestational age was estimated using the date of last menstrual period, which may overestimate premature births [22]. SGA was defined as a live born infant with birth weight below the 10th percentile for gender based on the INTERGROWTH-21st standards [23], which were applicable to gestational ages from 33–42 weeks. We extrapolated values for 30–32 weeks using the equations provided. We examined the association of vitamin D concentration and individual components of the composite adverse pregnancy outcome. The controls for stillbirth were live births regardless of prematurity status or SGA status. The controls for premature outcome were non-premature (34–42 weeks of gestation) regardless of SGA status. The controls for SGA were appropriate weight-for-gestational age regardless of prematurity status.

### 2.4. Statistical Analysis

In the primary analysis, unconditional logistic regression models were used to describe the association of 25(OH)D concentrations and the composite outcome [24,25,26]. The possibility of a non-linear relationship of 25(OH)D concentrations with the composite adverse pregnancy outcome was examined non-parametrically with restricted cubic splines [27]. A test for non-linearity was done using the likelihood ratio test which compared the model with only the linear term to the model with the linear and the cubic spline terms. Covariates for all multivariable analyses (including non-linear models) were selected based on prior knowledge of possible associations with individual adverse pregnancy outcomes. These included maternal age; number of previous pregnancies; body mass index (BMI) (weight (kg)/height (m)^2^) categorized as underweight (BMI < 18.5kg/m^2^), normal weight (BMI within 18.5–24.9 kg/m^2^) and overweight/obese (BMI ≥ 25.0 kg/m^2^); anaemia (defined as haemoglobin < 11.0 g/dL); and experimental regimen received (zinc and vitamin A supplements). Other socio-economic variables with *p*-value < 0.25 in univariable models were included.

Secondary analysis of individual components of the composite outcome used weighted logistic regression models [28,29]. We sought to produce a control sample for each component that would resemble a control sample chosen for that component in an independent analysis. Since we had a complete sample of the other components of the composite outcome, those who had experienced one or more of three secondary outcomes were oversampled relative to the original controls. We therefore down weighted them so that the sum of the weights would equal the number expected to be chosen if the sampling proportions had been the same as for the original controls. For each individual outcome, cases received unit weight and controls received outcome specific weight (non-premature SGA, non-premature non-SGA, premature non-SGA, and premature-SGA) equal to the ratio of the selection probabilities in the main analysis and the current study sample. The Firth penalized maximum likelihood estimation method for logistic regression model was adopted in the presence of complete separation of data points for all analysis of individual component outcomes [30,31,32]. Due to low prevalence of vitamin D deficiency, statistical analysis for individual adverse pregnancy outcomes did not use the three level vitamin D classification as defined by the IOM except for descriptive analysis. To investigate possible bias induced by missing vitamin D concentration for premature birth <30 weeks of gestation, sensitivity analysis was done using multiple imputation techniques with 10 imputed dataset [33]. The imputation of missing 25(OH)D was done using full conditional specification [FCS] method [34], and actual weeks of gestation was used in this model. Results were considered statistically significant if the 2-sided *p*-value was <0.05. Data management and data analysis were done in SAS version 9.4 (SAS Institute Inc., Cary, NC, USA).

Ethical approvals were obtained from the Harvard T.H. Chan School of Public Health Institutional Review Board, the Muhimbili University of Health and Allied Sciences (MUHAS) Senate of Research and Publications Committee, Tanzania’s National Institute for Medical Research, and the University Health Network (Toronto, ON, Canada). The IRB approval code for Harvard is 18,573, the MUHAS is Ref.No.2015-06-26/AEC/Vol.IX/119 and the University Health Network is 14-7313.

## 3. Results

### 3.1. Participants

**Figure 1:**
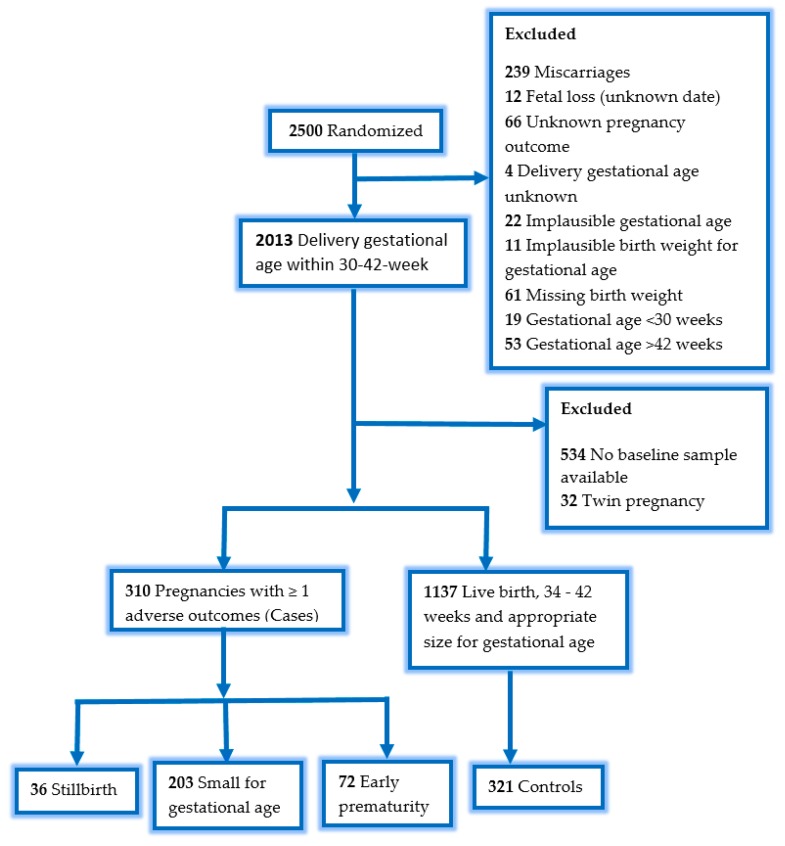
Sample flowchart, one pregnancy had premature birth and small for gestational age birth.

Among 2500 pregnant women in the parent trial, 487 were excluded because of miscarriages, fetal losses of unknown date, unknown pregnancy outcome, unknown delivery gestational age, implausible gestational age, implausible birth weight for gestational age, missing birth weight, gestational age < 30 weeks or > 42 weeks (Figure 1). Of the remaining 2013 women, 534 women did not have a baseline plasma sample available for 25(OH)D concentration analysis and 32 women had twin pregnancy. Of the remaining participants, 310 women had one or more adverse pregnancy outcome and were considered cases: 36 had stillbirths, 72 had premature live births, and 203 had SGA live births (including one participant who had both premature and SGA birth). Among the 203 SGA, 194 (96%) were term (birth > 37 weeks) births. We randomly selected 321 women from women with live, non-premature births with birth weight appropriate for gestational age as controls.

The average gestational age at baseline was 10 (standard deviation (SD): 2.4) weeks and was similar between cases and controls (Table 1). The majority of participants were ≤24 years, unemployed, married or cohabiting, with 0–7 years of education, and classified as normal BMI. This was similar for individual adverse pregnancy outcomes (Table S1).

### 3.2. The Association of Vitamin D Concentration and the Composite Adverse Pregnancy Outcomes

In controls, 5(2%) were vitamin D deficient (<20.0 ng/mL), 17(5%) had insufficient vitamin D concentration (20.0–29.9 ng/mL) and 299(93%) had sufficient vitamin D concentration (25(OH) ≥ 30.0 ng/mL). The average 25(OH)D concentrations were 42.3 (SD: 8.0) ng/mL in the cases and 42.7 (SD: 8.6) ng/mL in the controls. Pregnant women with 25(OH)D concentrations classified in second tertile (40.10–46.71 ng/mL) had slightly high proportions of the composite outcome (37%) compared to controls (31%) (Table S2). There was no association between 25(OH)D concentrations and having the composite adverse pregnancy outcome. Compared to women with sufficient 25(OH)D concentrations, women with 25(OH)D < 20.0 ng/mL had 1.82 times the odds of having the outcome (OR = 1.82, 95% CI: 0.56–5.93; *p* = 0.32) (Table 2).

**Figure 2:**
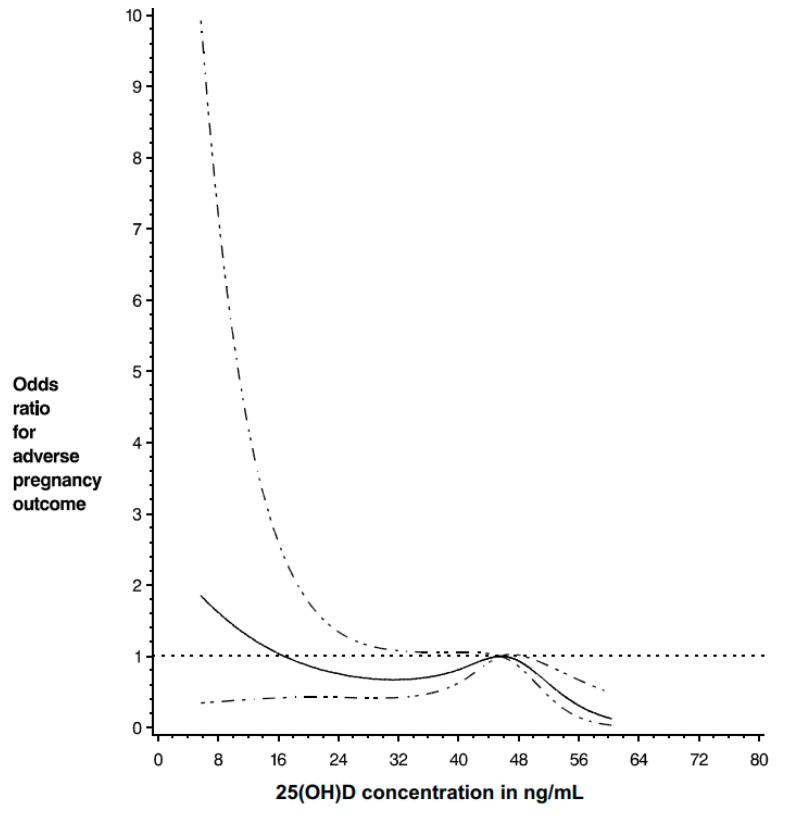
The adjusted relationship of 25(OH)D concentration and the composite adverse pregnancy outcome. The model included maternal age, employment status, wealth quartile, number of previous pregnancies, zinc supplement, vitamin A supplement, body mass index and anaemia status.

However, the results showed a non-linear association between first trimester 25(OH)D concentrations and the composite adverse pregnancy outcome (*p* = 0.02, Figure 2). A higher risk of the composite adverse pregnancy outcome was observed at low levels of 25(OH)D concentrations (25(OH)D < 20.0 ng/mL), but the confidence intervals were wide due to few data points. High levels of 25(OH)D concentrations (25(OH)D > 48 ng/mL) were associated with reduced odds of the composite adverse pregnancy outcome.

### 3.3. The Association of Vitamin D Concentration and Individual Adverse Pregnancy Outcomes

Among women who experienced stillbirth, 14% had 25(OH)D concentrations < 30.0 ng/mL and 6% had 25(OH)D concentrations < 20.0 ng/mL; the corresponding proportions for controls were 6% and 2% (Table 3).

There was a 3-fold increased odds of stillbirth in women with 25(OH)D concentrations < 30.0 ng/mL during early pregnancy (OR = 3.11, 95% CI: 1.18–8.23, *p* = 0.02) (Table 4). Similarly, women in the first tertile of 25(OH)D concentrations (5.71–40.03 ng/mL) had an increased odds of stillbirth compared to women in third tertile (46.73–60.35 ng/mL) (OR = 2.46, 95% CI: 1.12–5.43, *p* = 0.03) (Table S3). We did not note significant associations of 25(OH)D concentrations during early pregnancy and premature or SGA births.

## 4. Discussion

Vitamin D deficiency was not common in these mostly young, low parity women in urban Tanzania. We noted there was a non-linear association of first trimester 25(OH)D concentration and the composite adverse pregnancy outcome of stillbirth, premature, or SGA births, in which a protective association was observed from 25(OH)D concentration above 48 ng/mL. The 25(OH)D concentration was also associated with stillbirth as an individual outcome.

The association of stillbirth and low 25(OH)D concentration contrasts with findings in Bangladesh [13] and China [35], where no such association was observed. However, these studies differ from our study in terms of gestational age at which 25(OH)D concentration was assessed. The Bangladesh and China studies assessed 25(OH)D concentration at 17–24 weeks of gestation and 16–20 weeks of gestation, respectively, compared to 4–13 weeks in the current study. Our results also conflict with a study which assessed 25(OH)D concentration in the first trimester (10–14 week of gestation) among Australian women in a sun-rich latitude where vitamin D supplementation was uncommon; the findings did not support the association of maternal vitamin D status and stillbirth [17]. We speculate that geographical location and socioeconomic factors may explain the discrepancies observed between the study and ours despite similarities in prevalence of vitamin D deficiency. Hollis and Wagner [36], suggested that during early pregnancy, vitamin D prevents fetal rejection by the mother through its immunomodulatory effects, providing a possible mechanism that could explain the association of increased risk of stillbirths among women with low 25(OH)D concentrations.

It is postulated that vitamin D during early pregnancy has a role in implantation, placentation and maintenance of health pregnancy [37]. In normal early pregnancy, vitamin D receptors increases significantly. It has been suggested that a decrease in placental vitamin D receptors contributes to an increase in apoptosis which is associated with fetal growth retardation pathogenesis [37]. This mechanism is biologically plausible, but unlike others [7,10,16,38] we did not find an evidence for the association of 25(OH)D concentrations and SGA births.

A cohort study conducted in China which assessed SGA births in term infants found increased risk of SGA births in women with vitamin D deficiency during the first prenatal visit [10]. However, most other studies were conducted in high latitude countries where the prevalence of vitamin D deficiency during pregnancy is high due to limited sun light and long winter seasons, compared to tropical countries. This geographical heterogeneity may contribute to conflicting results in these studies. It has also been argued that null findings in these studies stem from methodological problems, small sample size, or assessment of 25(OH)D concentration late in pregnancy [10]. Our study is notable because unlike most previous studies, we assessed 25(OH)D concentrations in the first trimester.

We found no an association of 25(OH)D concentration during early pregnancy and prematurity. This finding was inconsistent with a large study conducted in the USA [39] which reported a 2-fold increased risk of premature births (births < 34 weeks of gestation) in women with 25(OH)D < 20.0 ng/mL. In addition, McDonnell et al. [40], found maternal 25(OH)D concentration ≥ 40.0 ng/mL (versus < 40.0 ng/mL) was associated with 60% reduced risk of premature births (birth < 37 weeks of gestations). We also examined the association of premature (birth < 34 weeks of gestation) and concentration < 40.0 ng/mL and we did not observe an association. Since 25(OH)D concentration during early pregnancy has a role of immunomodulatory effects [36,41], infection and inflammation during pregnancy contributes to prematurity [41], it is thought that sufficient 25(OH)D concentration during pregnancy will contribute to protect premature births through its anti-inflammatory activities [41]. However, our results do not support this hypothesized mechanism.

As we noted above, various cut-off points of 25(OH)D concentration exist in the literature. Low 25(OH)D concentration has been assessed using cut-off points ranging from <12.0 ng/mL up to <40.0 ng/mL. To some extent this could explain the contradicting findings observed in previous studies. We also noted that the prevalence of low 25(OH)D concentration was dependent on cut-off points implemented (7% for 25(OH)D < 30.0 ng/mL and 33% for 25(OH)D < 40.0 ng/mL). These variations highlight the need for a universally adopted 25(OH)D concentration classification for pregnant women. Nevertheless, we noted that the association between 25(OH)D concentration during pregnancy and adverse pregnancy outcomes did not substantially differ with different cut-offs.

Using 25(OH)D concentrations of <20.0 ng/mL and <30.0 ng/mL to define insufficiency, we found the prevalence of low 25(OH)D concentration was uncommon, in contrast to estimates obtained from a comparable HIV-negative population in Kenya [42]. HIV status may also play role in determining prenatal 25(OH)D concentrations. A study done among HIV positive pregnant women in Dar-es-Salaam, Tanzania found high prevalence (39%) of low vitamin D concentrations (25(OH)D < 32.0 ng/mL) at 12–27 weeks of gestation [12].

Strengths of the study include being among the first large and appropriately analyzed study in Africa to investigate the association of first trimester 25(OH)D concentrations and the composite adverse pregnancy outcomes as well as individual adverse pregnancy outcomes. We were able to show a non-linear association of the first trimester 25(OH)D concentrations and the composite adverse pregnancy outcomes. We also assessed the association of individual pregnancy outcomes given that the burden of these adverse outcomes is still high.

The study has several limitations worth noting. Gestational age was estimated based on date of last menstrual period, the approach may overestimate [22] or underestimate [43] the occurrence of premature births. However, a large study conducted in the USA comparing gestational age based on last menstrual period and clinically estimated gestational age (ultrasound) showed similar results by both methods [15]. In Tanzania ultrasound is not routinely available. While misclassification of premature births may have occurred, this is likely to be non-differential with respect to 25(OH)D concentrations. To reduce the effect of misclassification in the definition of prematurity, we defined prematurity as births occurring before 34 weeks of gestation. We could not investigate the effect of vitamin D concentration on other conditions related to prematurity such as preeclampsia as data on these outcomes were not available. Further, the study was restricted to women who delivered between 30–42 weeks of gestation, which could have attenuated our results if those who delivered before 30 weeks were more likely to be vitamin D deficient. Only 11 women among those who delivered before 30 weeks had a baseline sample, however sensitivity analysis done using multiple imputation techniques showed consistent findings with the primary findings. 

## 5. Conclusions

Maternal nutritional factors associated with increased risk of stillbirths and other adverse pregnancy outcomes such as premature births and SGA births are crucial to examine, given the burden and long term effects of these outcomes. In this study, we noted the non-linear association of 25(OH)D concentration during early pregnancy and the composite of adverse pregnancy outcomes of stillbirth, SGA birth and premature birth. We also noted a significantly increased risk of stillbirths in women with low 25(OH)D concentration during early pregnancy. Vitamin D concentration in early pregnancy may be an important factor in determining the course of pregnancy, and further research is warranted to examine whether this association is causal.

## Figures and Tables

**Table 1:** Baseline maternal characteristics among cases of adverse pregnancy outcomes and controls in Dar-es-Salaam, Tanzania.

Characteristics	Cases (Either Stillbirth ^1^, Premature ^2^ or SGA ^3^)	Controls
Overall, n	310	321
Vitamin D concentration in ng/mL, mean (SD)	42.3 (8.0)	42.7 (8.6)
**Vitamin D concentration in ng/mL, n (%)**		
Sufficient (≥30.0 ng/mL)	289 (93.2)	299 (93.2)
Low level (<30.0 ng/mL)	21 (6.8)	22 (6.8)
**Vitamin D concentration in ng/mL, n (%)**		
Sufficient (≥30.0 ng/mL)	289 (93.2)	299 (93.2)
Insufficient (20.0–29.9 ng/mL)	14 (4.5)	17 (5.3)
Deficient (<20.0 ng/mL)	7 (2.3)	5 (1.5)
**Vitamin D concentration in ng/mL, n (%)**		
Sufficient (≥40.0 ng/mL)	211(68.1)	213 (66.4)
Low level (<40.0 ng/mL)	99 (31.9)	108 (33.6)
Maternal age in completed years, mean (SD)	22.7 (4.3)	22.4 (3.6)
**Maternal age in years, n (%)**		
≤24	221 (72.0)	252 (78.5)
25–34	79 (25.7)	66 (20.6)
≥35	7 (2.3)	3 (0.9)
Gestational age in weeks, mean(SD) ^4^	9.9 (2.3)	9.8 (2.4)
**Employment, n (%)**		
Employed ^5^	85 (27.5)	95 (29.8)
Unemployed	187 (60.5)	175 (54.9)
Other	37 (12.0)	49 (15.3)
**Marital status, n (%)**		
Living single ^6^	31 (10.0)	37 (11.5)
Married or cohabitating	278 (90.0)	284 (88.5)
**Years of education, n (%)**		
0–7	234 (75.5)	225 (70.1)
8–11	66 (21.3)	78 (24.3)
≥12	10 (3.2)	18 (5.6)
**Wealth quartile, n (%)**		
1 (Lowest)	67 (22.6)	71 (23.8)
2	64 (21.6)	82 (27.5)
3	103 (34.8)	89 (29.9)
4 (Highest)	62 (21.0)	56 (18.8)
**Number of previous pregnancies, n (%)**		
First	169 (54.5)	135 (42.1)
Second	141 (45.5)	186 (57.9)
**Vitamin A supplements, n (%)**		
Not received	153 (49.4)	170 (53.0)
Received	157 (50.6)	151 (47.0)
**Zinc supplements, n (%)**		
Not received	154 (49.7)	158 (49.2)
Received	156 (50.3)	163 (50.8)
Body mass index (kg/m^2^), mean (SD)	22.4 (3.9)	23.1 (4.4)
**Body mass index (kg/m^2^), n (%)**		
Underweight	37 (12.0)	32 (10.0)
Normal	213 (69.2)	213 (66.6)
Overweight or obese	58 (18.8)	75 (23.4)
Haemoglobin in g/dL, mean (SD)	11.4 (1.6)	11.5 (1.4)
**Anaemia status, n (%)**		
Normal Hb (≥11.0 g/dL)	199 (65.7)	221 (70.8)
Anaemic (˂11.0 g/dL)	104 (34.3)	91 (29.2)

^1^ Stillbirth does not include miscarriage. ^2^ Premature delivery in this analysis does not include premature stillbirth and early premature (birth before 30 weeks). ^3^ Small for gestational age as defined by below 10th percentile based on Intergrowth standards. ^4^ Gestational age at enrollment based on last menstrual period. ^5^ Employed include skilled, unskilled and informal employment. ^6^ Living single includes never married, divorced, separated and widow.

**Table 2:** Relationship of composite adverse pregnancy outcomes and vitamin D concentration during early pregnancy, Dar-es-Salaam, Tanzania ^1^.

Exposure of Interest	Unadjusted OR ^2^ [95% CI ^3^]	*p*-Value	Adjusted OR ^2^ [95% CI ^3^]	*p*-Value
**Vitamin D concentration in ng/mL**				
Sufficient (≥30.0 ng/mL)	Ref		Ref	
Low (<30.0 ng/mL)	0.99 [0.53, 1.84]	0.97	1.05 [0.55, 1.98]	0.89
**Vitamin D concentration in ng/mL**				
Sufficient (≥30.0 ng/mL)	Ref		Ref	
Insufficient (20.0–29.9 ng/mL)	0.85 [0.41, 1.76]	0.67	0.84 [0.39, 1.78]	0.64
Deficient (<20.0 ng/mL)	1.45 [0.46, 4.62]	0.53	1.82 [0.56, 5.93]	0.32
**Vitamin D concentration ng/mL**				
Sufficient (≥40.0 ng/mL)	Ref		Ref	
Low (<40.0 ng/mL)	0.93 [0.66, 1.29]	0.65	0.90 [0.63, 1.27]	0.54

^1^ Adjusted models included maternal age, employment status, wealth quartile, number of previous pregnancies, zinc supplement, vitamin A supplement, body mass index and anaemia status. ^2^ OR is odds ratio. ^3^ CI is confidence interval.

**Table 3:** Distribution of vitamin D concentration among cases and control for individual pregnancy outcome, Dar-es-Salaam, Tanzania.

Characteristics	Stillbirth	Controls	Small for Gestation Age among Live Births ^1^	Controls	Premature (Birth before 34 Weeks) among Live Births ^2^	Controls
Overall, n	36	595	203	392	72	523
Vitamin D concentration in ng/mL, mean (SD)	39.5 (10.0)	42.7 (8.2)	42.1 (8.2)	43.0 (8.2)	44.4 (5.6)	42.5 (8.5)
**Vitamin D concentration in ng/mL, n (%)**						
Sufficient (≥30.0 ng/mL)	31 (86.1)	557 (93.6)	188 (92.6)	369 (94.1)	71 (98.6)	486 (92.9)
Low (<30.0 ng/mL)	5 (13.9)	38 (6.4)	15 (7.4)	23 (5.9)	1 (1.4)	37 (7.1)
**Vitamin D concentration in ng/mL, n (%)**						
Sufficient (≥30.0 ng/mL)	31 (86.1)	557 (93.6)	188 (92.6)	369 (94.1)	71 (98.6)	486 (92.9)
Insufficient (20.0–29.9 ng/mL)	3 (8.3)	28 (4.7)	10 (4.9)	18 (4.6)	1 (1.4)	27 (5.2)
Deficient (<20.0 ng/mL)	2 (5.6)	10 (1.7)	5 (2.5)	5 (1.3)	0	10 (1.9)
**Vitamin D concentration in ng/mL, n (%)**						
Sufficient (≥40.0 ng/mL)	18 (50.0)	406 (68.2)	137 (67.5)	269 (68.6)	57 (79.2)	349 (66.7)
Low (<40.0 ng/mL)	18 (50.0)	189 (31.8)	66 (32.5)	123 (31.4)	15 (20.8)	174 (33.3)

^1^ Small for gestational age was defined as birth weight below 10th percentile based on Intergrowth standards. ^2^ Premature delivery in this analysis does not include birth before 30 weeks.

**Table 4:** Relationships of individual adverse pregnancy outcomes and vitamin D concentration during early pregnancy, Dar-es-Salaam, Tanzania ^1^.

Exposure of Interest	Unadjusted	*p*-Value	Adjusted	*p*-Value
	OR ^2^ [95% CI ^3^]		OR ^2^ [95% CI ^3^]	
**Stillbirth**				
Vitamin D concentration in ng/mL				
Sufficient (≥30.0 ng/mL)	Ref		Ref	
Low (<30.0 ng/mL)	2.27 [0.84, 6.15]	0.11	3.11 [1.18, 8.23]	0.02
Vitamin D concentration in ng/mL				
Sufficient (≥40.0 ng/mL)	Ref		Ref	
Low (<40.0 ng/mL)	2.05 [1.04, 4.03]	0.04	2.53 [1.31, 4.89]	0.01
**Premature (birth before 34 weeks) ^4^**				
Vitamin D concentration in ng/mL				
Sufficient (≥30.0 ng/mL)	Ref		Ref	
Low (<30.0 ng/mL)	0.19 [0.03, 1.40]	0.10	0.29 [0.06, 1.50]	0.14
Vitamin D concentration in ng/mL				
Sufficient (≥40.0 ng/mL)	Ref		Ref	
Low (<40.0 ng/mL)	0.52 [0.29, 0.95]	0.03	0.59 [0.32, 1.07]	0.08
**Small for gestational age ^5^**				
Vitamin D concentration in ng/mL				
Sufficient (≥30.0 ng/mL)	Ref		Ref	
Low (<30.0 ng/mL)	1.15 [0.61, 2.16]	0.67	1.33 [0.69, 2.56]	0.39
Vitamin D concentration in ng/mL				
Sufficient (≥40.0 ng/mL)	Ref		Ref	
Low (<40.0 ng/mL)	0.99 [0.70, 1.39]	0.93	0.94 [0.65, 1.35]	0.72

^1^ Adjusted models included maternal age, employment status, wealth quartile, zinc supplement, vitamin A supplement, number of previous pregnancies, body mass index, and anaemia status. ^2^ OR is odds ratio. ^3^ CI is confidence interval. ^4^ Premature delivery in this analysis does not include birth before 30 weeks. ^5^ Small for gestational age is defined as birth weight below the 10th percentile based on Intergrowth standards.

## Supplementary Materials

The following are available online at https://www.mdpi.com/2072-6643/11/12/2906/s1, Table S1: Distribution of baseline characteristics for each individual pregnancy outcome, Dar-es-Salaam, Tanzania. Table S2: The distribution of vitamin D concentration tertiles among cases of adverse pregnancy outcomes and controls in Dar-es-Salaam, Tanzania Table S3: Relationships of the composite and individual adverse pregnancy outcomes and vitamin D concentration tertiles during early pregnancy, Dar-es-Salaam, Tanzania.
