# Supplementary material for: Vitamin D Concentration during Early Pregnancy and Adverse Outcomes among HIV-Negative Women in Dar-es-Salaam, Tanzania: A Case-Control Study
Source: Nutrients. 2019 Dec 2;11(12):2906. doi: 10.3390/nu11122906 (PMC6949980; doi:10.3390/nu11122906)
Supplement: Supplementary file 1 [file nutrients-11-02906-s001.pdf]

Supplementary Materials

Table S1: Distribution of baseline characteristics for each individual pregnancy outcome, Dar-es-Salaam, Tanzania.

Characteristics	Stillbirth	Controls	Small for gestation age among live births [1]	Controls	Premature (birth before 34 weeks) among live births [2]	Controls
Overall, n	36	595	203	392	72	523
Vitamin D concentration in ng/mL tertiles, n (%)						
First (5.71 - 40.03 ng/mL)	18 (50.0)	191 (32.1)	66 (32.5)	125 (31.9)	16 (22.2)	175 (33.4)
Second (40.10 - 46.71 ng/mL)	9 (25.0)	206 (34.6)	75 (37.0)	131 (33.4)	32 (44.4)	174 (33.3)
Third (46.73 - 60.35 ng/mL)	9 (25.0)	198 (33.3)	62 (30.5)	136 (34.7)	24 (33.3)	174 (33.3)
Maternal age in years, mean (SD)	23.0 (3.1)	22.5 (4.0)	22.9 (4.4)	22.3 (3.8)	22.0 (4.6)	22.6 (3.9)
Maternal age in years, n (%)						
≤ 24	26 (72.2)	447 (75.5)	137 (68.5)	310 (79.1)	58 (81.7)	389 (74.7)
25-34	10 (27.8)	135 (22.8)	58 (29.0)	77 (19.6)	11 (15.5)	124 (23.8)
≥ 35	0	10 (1.7)	5 (2.5)	5 (1.3)	2 (2.8)	8 (1.5)
Gestational age in weeks, mean(SD)[3]	9.9 (2.5)	9.8 (2.4)	10.0 (2.4)	9.7 (2.4)	9.5 (2.3)	9.9 (2.4)
Employment, n (%)						
Employed[4]	7 (19.4)	173 (29.2)	51 (25.2)	122 (31.3)	27 (37.5)	146 (28.1)
Unemployed	23 (63.9)	339 (57.3)	124 (61.4)	215 (55.1)	41 (56.9)	298 (57.3)
Other	6 (16.7)	80 (13.5)	27 (13.4)	53 (13.6)	4 (5.6)	76 (14.6)
Marital status, n (%)						
Living single[5]	2 (5.7)	66 (11.1)	18 (8.9)	48 (12.2)	12 (16.7)	54 (10.3)
Married or cohabitating	33 (94.3)	528 (88.9)	185 (91.1)	344 (87.8)	60 (83.3)	469 (89.7)
Years of education, n (%)						
0 - 7	27 (75.0)	432 (72.6)	151 (74.4)	281 (71.7)	57 (79.2)	375 (71.7)
8 - 11	8 (22.2)	136 (22.9)	44 (21.7)	92 (23.5)	14 (19.4)	122 (23.3)
≥ 12	1 (2.8)	27 (4.5)	8 (3.9)	19 (4.8)	1 (1.4)	26 (5.0)
Wealth quartile, n (%)						
1 (Lowest)	4 (12.5)	134 (23.8)	52 (26.8)	82 (22.3)	11 (15.5)	123 (25.0)
2	9 (28.1)	137 (24.4)	44 (22.7)	93 (25.3)	11 (15.5)	126 (25.7)
3	12 (37.5)	180 (32.0)	67 (34.5)	113 (30.7)	25 (35.2)	155 (31.6)

4 (Highest)	7 (21.9)	111 (19.8)	31 (16.0)	80 (21.7)	24 (33.8)	87 (17.7)
Number of previous pregnancies, n (%)						
First	18 (50.0)	286 (48.1)	110 (54.2)	176 (44.9)	41 (56.9)	245 (46.9)
Second	18 (50.0)	309 (51.9)	93 (45.8)	216 (55.1)	31 (43.1)	278 (53.1)
Vitamin A supplements, n (%)						
Not received	21 (58.3)	302 (50.8)	101 (49.8)	201 (51.3)	31 (43.1)	271 (51.8)
Received	15 (41.7)	293 (49.2)	102 (50.2)	191 (48.7)	41 (56.9)	252 (48.2)
Zinc supplements, n (%)						
Not received	15 (41.7)	297 (49.9)	102 (50.2)	195 (49.7)	38 (52.8)	259 (49.5)
Received	21 (58.3)	298 (50.1)	101 (49.8)	197 (50.3)	34 (47.2)	264 (50.5)
Body mass index (kg/m^2), mean (SD)	23.8 (3.8)	22.7 (4.2)	22.2 (4.0)	23.0 (4.3)	22.3 (3.4)	22.8 (4.3)
Body mass index (kg/m^2), n (%)						
Underweight	1 (2.8)	68 (11.5)	27 (13.4)	41 (10.5)	9 (12.7)	59 (11.3)
Normal	25 (69.4)	401 (67.7)	141 (69.8)	260 (66.7)	48 (67.6)	353 (67.8)
Overweight or obese	10 (27.8)	123 (20.8)	34 (16.8)	89 (22.8)	14 (19.7)	109 (20.9)
Haemoglobin in g/dL, mean (SD)	11.3 (1.5)	11.5 (1.5)	11.6 (1.5)	11.4 (1.4)	10.9 (1.5)	11.5 (1.5)
Anaemia status, n (%)						
Normal Hb (≥ 11.0 g/dL)	21 (58.3)	399 (68.9)	139 (70.6)	260 (68.1)	40 (56.3)	359 (70.7)
Anaemic (< 11.0 g/dL)	15 (41.7)	180 (31.1)	58 (29.4)	122 (31.9)	31 (43.7)	149 (29.3)

[1] Small for gestational age was defined as birth weight below 10th percentile based on Intergrowth standards.
[2] Premature delivery in this analysis does not include early premature (birth before 30 weeks).
[3] Gestational age at enrollment based on last menstrual period.
[4] Employed include skilled, unskilled and informal employment.
[5] Living single includes never married, divorced, separated and widow.

Table S2: The distribution of vitamin D concentration tertiles among cases of adverse pregnancy outcomes and controls in Dar-es-Salaam, Tanzania.

Characteristics	Cases (Either stillbirth [1], premature [2] or SGA [3])	Controls
Overall, n	310	321
Vitamin D concentration tertile in ng/mL, n (%)		
First (5.71 - 40.03 ng/mL)	100 (32.3)	109 (34.0)
Second (40.10 - 46.71 ng/mL)	115 (37.1)	100 (31.1)
Third (46.73 - 60.35 ng/mL)	95 (30.6)	112 (34.9)

[1] Stillbirth does not include miscarriage.

[2] Premature delivery in this analysis does not include premature stillbirth and early premature (birth before 30 weeks).

[3] Small for gestational age as defined by below 10th percentile based on Intergrowth standards.

[4] Gestational age at enrollment based on last menstrual period.

Table S3: Relationships of the composite and individual adverse pregnancy outcomes and vitamin D concentration tertiles during early pregnancy, Dar-es-Salaam, Tanzania[1]

Exposure of interest	Unadjusted OR[2] [95% CI[3]]	P-value	Adjusted OR[2] [95% CI[3]]	P-value
Composite outcome [4]				
Vitamin D concentration in ng/mL tertiles				
First (5.71 – 40.03 ng/mL)	1.08 [0.74, 1.59]	0.69	1.05 [0.70, 1.58]	0.80
Second (40.10 – 46.71 ng/mL)	1.36 [0.93, 1.99]	0.12	1.36 [0.92, 2.03]	0.13
Third (46.73 – 60.35 ng/mL)	Ref		Ref	
Stillbirth				
Vitamin D concentration tertile in ng/mL				
First (5.71 - 40.03 ng/mL)	2.06 [0.91, 4.70]	0.08	2.46 [1.12, 5.43]	0.03
Second (40.10 - 46.71 ng/mL)	1.05 [0.41, 2.69]	0.93	1.05 [0.44, 2.53]	0.91
Third (46.73 - 60.35 ng/mL)	Ref		Ref	
Premature (birth before 34 weeks) [5]				
Vitamin D concentration tertile in ng/mL				
First (5.71 - 40.03 ng/mL)	0.68 [0.35, 1.31]	0.25	0.75 [0.39, 1.47]	0.40
Second (40.10 - 46.71 ng/mL)	1.43 [0.81, 2.51]	0.22	1.44 [0.81, 2.58]	0.22
Third (46.73 - 60.35 ng/mL)	Ref		Ref	
Small for gestational age [6]				
Vitamin D concentration tertile in ng/mL				
First (5.71 - 40.03 ng/mL)	1.12 [0.74, 1.67]	0.60	1.06 [0.69, 1.61]	0.79
Second (40.10 - 46.71 ng/mL)	1.32 [0.89, 1.96]	0.17	1.32 [0.88, 1.99]	0.18
Third (46.73 - 60.35 ng/mL)	Ref		Ref	

[1] Adjusted models included maternal age, employment status, wealth quartile, zinc supplement, vitamin A supplement, number of previous pregnancies, body mass index, and anaemia status.

[2] OR is odds ratio.

[3] CI is confidence interval.

[4] The composite of stillbirths, premature births before 34 weeks and SGA births

[5] Premature delivery in this analysis does not include birth before 30 weeks.

[6] Small for gestational age is defined as birth weight below the 10th percentile based on Intergrowth standards.
